# Traité Des Maladies Mentales, Considerées Sous Les Rapports Medical, Hygiénique, Et Medico-legal

**Published:** 1838-08-01

**Authors:** 


### BIBLIOGRAPHICAL NOTICE.

Traité des Maladies Mentales, *Considerées sous les Rapports Medical, Hygiénique, et Medico-legal.* Par E. Esquirol, Medecin en chef de la maison royale des Aliénés de Charenton, &c., &c., 2 vol. 8vo. Paris, 1838.

M. Esquirol has been, for many years, constantly occupied with the study and treatment of mental diseases; his reputation has increased with the progress of knowledge, and has become more firmly established, since the increase in the number of physicians specially occupied with the treatment of the insane. The memoirs which he published, from time to time, in the Dictionnaire des Sciences Medicales, have lost none of their freshness, for truth never fades; and, although these essays may seem incomplete, and sometimes imperfect, they will remain as part of the history of classical medicine, for they were written in the spirit of true inductive observation, directed by enthusiastic devotion to the interests of the unfortunate insane.

The present work is not strictly new; it consists of a series of those essays, which must be familiar to a portion of our readers; but they have all been carefully revised, and are, as it were, sanctioned by this republication, in a collective form. They are thrown into as regular an order as possible, and are not very different from a systematic work. A succession of essays can never, however, completely replace an elaborate work, and we cannot avoid joining in the regret, which M. Esquirol himself expresses, that his numerous engagements should prevent him from exerting his long promised resolution of publishing a complete account of his experience in the management of insanity. We regret the loss, without being surprised at it, for few physicians, who have collected much instructive and useful knowledge, in a long course of observation, succeed in rendering that knowledge available to the world, if they postpone its publication until the decline of life. Advancing age brings with it increasing infirmities, and professional engagements of a more lucrative kind, than those which occupy the earlier years of a physician. The hours which remain after the daily labours of practice, can scarcely be devoted to toils, which are more severe and less profitable; and after years of anticipation, a physician usually ends by relinquishing his promised work.

The arrangement of the work is as nearly similar to that of a systematic treatise as possible. A memoir upon insanity, which was published more than twenty years since in the Dictionnaire de Médecine serves as an introduction to the treatises on the particular varieties of insanity which compose the body of the work. Hence the reader is enabled to observe something like a systematic course of study, and is not rapidly hurried from one subject to another, as necessarily occurs with works consisting of a collection of treatises, written at different times, and on various diseases, not closely connected together.

If there is not sufficient unity in the plan and execution of the whole work, the most perfect simplicity of practice, and most conscientious observation of the symptoms of insanity are shown in each portion of it. This is one of the strongest characteristics of the work; there is not a single memoir in which the author does not show that he teaches what he believes, and that he believes because he has observed conscientiously, and without a desire to overturn established medical opinions, or rashly to introduce a system of practice which is to supersede all others in which our predecessors may have put faith. M. Esquirol is strongly impressed with the natural disposition, if we may use the word, of most diseases to a termination; hence he has devoted most of these memoirs to the study of the history of insanity; and the treatise on the crises of insanity is one of the most impressive in the work. This mode of termination of the disease is by no means rare; in our insane hospitals we have often witnessed it, sometimes in cases which had resisted the usual treatment.

There are numerous other modes of termination of insanity, either from the gradual and imperceptible changes occurring during the course of the disease, or from the action of our remedies. These remedies are made by M. Esquirol subordinate to the natural course, and, as it were, subsidiary to the natural mode of treatment. Hence, in all cases, more especially in chronic insanity, he carefully abstains from any sudden active interference; but at the entrance of a patient he places him under observation, gains his confidence by gentle and friendly treatment, and then watches until he can interfere with the best prospects of success. His remedies are always simple; he rarely prescribes blood-letting largely, nor does he use purgatives, except of the mildest kind. Baths, both general and local, especially the shower bath, seclusion, and appropriate moral treatment, are found sufficient remedies for many acute cases, and offer the best results in chronic forms of insanity. Still, we think M. Esquirol is rather too sparing in remedial agents in the management of acute insanity; for although there is a distinction well marked in most cases between insanity and phrenitis, the line is not equally well drawn in a considerable number of instances of violent insanity, especially those occurring during the hot months of the year. Such cases require treatment of a more active character than M. Esquirol has advised. It is in chronic insanity that the mild, soothing method proves most useful, especially that happy combination of moral and medicinal treatment which is pursued at the private asylum of M. Esquirol. These results are not, however, so favourable as those which are claimed by some of the institution of this country. Without doubting the accuracy of these statements we fear that they are sometimes fallacious. For with no disease is it more easy for the physician to commit errors in the estimate of his success than with insanity. The degree of mental strength necessary to constitute perfect sanity is, of course, extremely various, and can only be fairly tested after the lapse of a considerable period.

The question as to the increase of insanity is treated by M. Esquirol in one of the memoirs; to a limited extent, he admits the influence of the various political and religious excitements which have involved the world, and have spent their utmost violence upon France. The increase in the number of the insane is, however, rather apparent than real, and arises, as is remarked by the author, chiefly from the multiplication and better management of lunatic asylums. The friends of lunatics are, therefore, more willing to entrust them to these institutions, than to the wretched habitations to which these unfortunates were formerly consigned.

A large portion of the second volume is devoted to an account of the hospital at Charenton. Of this we may speak at a future period.

